# RETRACTION: A Meta‐Analysis of the Effect of Endoscopic Submucosal Dissection Compared with Gastrectomy on the Wound Infection in Early Stomach Cancer Subjects

**DOI:** 10.1111/iwj.70233

**Published:** 2025-02-11

## Abstract

**RETRACTION:** SunC. , LiuW. , JiangJ. , ZhangH. , WangP. , SunJ. , and SunA. , “A Meta‐Analysis of the Effect of Endoscopic Submucosal Dissection Compared with Gastrectomy on the Wound Infection in Early Stomach Cancer Subjects,” International Wound Journal 20, no. 6 (2023): 2087–2094, 10.1111/iwj.14078.36629038 PMC10333015

The above article, published online on 11 January 2023, in Wiley Online Library (http://onlinelibrary.wiley.com/), has been retracted by agreement between the journal Editor in Chief, Professor Keith Harding; and John Wiley & Sons Ltd. Following an investigation by the publisher, all parties have concluded that this article was accepted solely on the basis of a compromised peer review process. The editors have therefore decided to retract the article. The authors did not respond to our notice regarding the retraction.

**RETRACTION:** C. Sun , W. Liu , J. Jiang , H. Zhang , P. Wang , J. Sun , and A. Sun , “A Meta‐Analysis of the Effect of Endoscopic Submucosal Dissection Compared with Gastrectomy on the Wound Infection in Early Stomach Cancer Subjects,” International Wound Journal 20, no. 6 (2023): 2087–2094, 10.1111/iwj.14078.36629038 PMC10333015

The above article, published online on 11 January 2023, in Wiley Online Library (http://onlinelibrary.wiley.com/), has been retracted by agreement between the journal Editor in Chief, Professor Keith Harding; and John Wiley & Sons Ltd. Following an investigation by the publisher, all parties have concluded that this article was accepted solely on the basis of a compromised peer review process. The editors have therefore decided to retract the article. The authors did not respond to our notice regarding the retraction.

